# LRG1 Suppresses Migration and Invasion of Esophageal Squamous Cell Carcinoma by Modulating Epithelial to Mesenchymal Transition

**DOI:** 10.7150/jca.36189

**Published:** 2020-01-14

**Authors:** Ninggang Zhang, Yaqiong Ren, Yusheng Wang, Lei Zhao, Bin Wang, Nina Ma, Zhengxing Gao, Bangwei Cao

**Affiliations:** 1 Cancer Center, Beijing Friendship Hospital, Capital Medical University, Beijing 100050, China; 2 Shanxi Cancer Hospital Affiliated to Shanxi Medical University, No. 3 of Zhigong Xincun Street, Xinghualing District, Taiyuan, Shanxi 030013, China

**Keywords:** LRG1, Esophageal Squamous Cell Carcinoma, Migration, Invasion, Epithelial to Mesenchymal Transition, TGFβ signaling

## Abstract

**Background**: Esophageal squamous cell carcinoma (ESCC) is a common cancer with poor prognosis. The molecular pathogenesis underlying ESCC remains to be explored. Leucine-rich ɑ-2-glycoprotein 1 (LRG1) has been implicated in the pathogenesis of various cancer types, however its role in ESCC is unknown.

**Materials and Methods**: Data from the public database was analyzed to address the expression of LRG1 in ESCC. Gain-of-function studies were performed in select ESCC cell lines by over-expression or addition of recombinant LRG1, while loss-of-function studies achieved by small interfering RNA mediated knockdown. Wound healing and transwell assays were conducted to investigate ESCC cell migration and invasion upon manipulating LRG1 levels. Western blot and Immunofluorescence staining were used to examine the changes in epithelial to mesenchymal transition (EMT) and TGFβ signaling pathway.

**Results**: LRG1 mRNA levels were found to be significantly down-regulated in patients with ESCC as well as in several ESCC cell lines. Silencing of LRG1 promoted, while overexpression of LRG1 inhibited ESCC cell migration and invasion. In line with this, Silencing of LRG1 enhanced, while overexpression of LRG1 reduced TGFβ signaling and EMT of ESCC cells.

**Conclusion/Significance**: LRG1 suppresses ESCC cell migration and invasion via negative modulation of TGFβ signaling and EMT. Down-regulation of LRG1 in ESCC patients may favor tumor metastasis and disease progression.

## Introduction

Esophageal cancer is the eighth most common cancer worldwide with an incidence of estimated 480,000 new patients every year and the sixth leading cause of cancer-related deaths 1. There are two main subtypes of esophageal cancer, esophageal squamous cell carcinoma (ESCC) and esophageal adenocarcinoma (EAC). ESCC accounts for more than 90% of esophageal cancer and is the fourth leading cause of death from cancer in China 2, 3. Despite the advances in diagnosis and therapeutics, ESCC still carries a poor prognosis—the five-year survival rate is less than 25% 4. There remains a need to uncover the molecular pathogenesis underlying this disease 5, 6.

Leucine-rich ɑ-2-glycoprotein 1 (LRG1) was identified in 1977 in human serum as a secreted glycoprotein 7. LRG1 is a highly conserved member of the leucine-rich repeat (LRR) family of proteins 8, which are involved in protein-protein interactions, cell signal transduction, cell adhesion, DNA repair, immune response, etc. In line with this, LRG1 has been reported to play a role in inflammatory response 9, 10. While serum LRG1 levels might be used as an indicator for tumor diagnosis 11-13, the expression, impact and potential mechanisms of LRG1 on malignant carcinomas remains controversial. The expression levels of LRG1 were found to be upregulated in several types of carcinomas such as ovarian cancer and colorectal cancer, where it promotes tumor cell proliferation and invasion while inhibiting apoptosis 14-17, 29. However, there are also studies showing that LRG1 expression is down-regulated in tumors, inhibiting tumor cell proliferation while promoting apoptosis 18,19. The impact of LRG1 on cell migration and invasion appeared to be controversial even in the same tumor type, such as hepatocellular carcinoma (HCC). Some researchers found LRG1 could promote HCC cell migration 14, while others reported an inhibitory role of LRG1 on the migratory and invasive potential of HCC cells 18. Further studies are warranted to address such discrepancies and to explore the involvement of LRG1 in other cancer types.

Although LRG1 has been implicated in many carcinoma progression, the research on the expression levels and biological functions of LRG1 in ESCC had just begun 39. In this study, we first investigated the expression of LRG1 in ESCC patients by data mining, and then explored the biological function of LRG1 in ESCC. We demonstrate that LRG1 can promote the apoptosis of ESCC cell lines, moreover, LRG1 inhibit ESCC cell metastasis by reduce the epithelial to mesenchymal transition (EMT) via the TGFβ/SMAD signaling pathway.

## Materials and Methods

### Cell lines and LRG1 treatment

The human ESCC cell lines (TE1, KYSE30 and EC109), the normal esophageal phosphorous epithelial cell Het-1A, and the *hepatoma cell line* HepG2, were all purchased from American Type Culture Collection (ATCC, Rockville, MD, USA). TE1, EC109 and Het-1A were cultured in RPMI-1640 medium plus 10% fetal bovine serum (Corning, USA), KYSE30 and HepG2 were cultured in DMEM plus 10% fetal bovine serum. All of cell lines were maintained at 37℃ under a humidified atmosphere of 5% CO_2_. Recombinant LRG1 was purchased from Sino Biological (China, catalog #13371-HCCH) and added to the culture medium at concentrations of 25-500ng/mL for the indicated time before harvesting.

### siRNA knockdown

Three siRNAs against LRG1 (SR314597) were purchased from OriGene (USA), The siRNA oligo sequences (sense strand) are the following: #1: 5'-GCAACCCGCUUAACAAAUAAUCCTG-3'; #2: 5'-GCUACAUCUAGAAGGCAACAAAUTG-3'; #3: 5'-GCCUAAGCUCCAAGAAUUGCACC-3'). KYSE30 cells were seeded in 6-well plates 24h before transfection. When cells confluence reached 70%-80%, transfection of siRNA was performed using Lipofectamine 3000 (Invitrogen, USA) according to the manufacturer's protocol.

### LRG1 overexpression

EC-109 cells were similarly seeded and transfected with a LRG1-overexpressing vector pCMV-LRG1 (SyngenTech, China) or the empty pCMV vector (SyngenTech, China) as control.

### RNA extraction and quantitative real-time RT-PCR

The total RNA was extracted from cultured cells using TRIzol reagent (Invitrogen, USA), according to the manufacturer's protocol, and cDNA was synthesized using the PrimeScript^TM^ RT Reagent Kit (Promega, Madison, WI, USA). Q-PCR of LRG1 mRNA expression was conducted using a FAST SYBR^TM^ Green Master Mix (Thermo Fisher Scientific, USA) on an ABI Prism 7700 Sequence Detector (Applied Biosystems, USA). GAPDH was used as internal control. The 2(-∆∆Ct) method was used for data analysis. The primers are as follows: LRG1, forward: 5'-GGACACCCTGGTATTGAAAGAAA-3'; reverse: 5'-TAGCCGTTCTAATTGCAGCGG-3'; GAPDH, forward: 5'-GGAGCGAGATCCCTCCAAAAT-3': reverse: 5'-GGCTGTTGTCATACTTCTCATGG-3'.

### Western blot analysis

The total proteins of ESCC cell lines were extracted using RIPA protein extraction reagent (Thermo Scientific, USA). The protein concentration was measured using the BCA assay (Thermo Scientific, USA). Equal amounts of proteins (30 μg/well) were separated on 10% SDS polyacrylamide gels and transferred to PVDF membranes (Millipore, USA). After blocking with 5% fat-free milk in TBST buffer for 30 minutes at room temperature, the membranes were incubated with primary antibodies overnight at 4℃. The membranes were then incubated with HRP-conjugated secondary antibody (Promega, USA) at RT for 1h. The protein bands were detected using chemiluminescence (Millipore, USA) and exposed to X-ray films. See supplementary Table 1 for antibody information.

### Wound-healing assay

ESCC cells were plated in 6-well plates to reach 90% confluence. The samples were then scratched manually using a pipette tip. After removal of cell debris by washing 3 times with phosphate-buffered saline (PBS), the wounded cell samples were covered with serum-free culture medium. Images were acquired at 0 and 24h post-scratching. The gap distances were measured using the Image J software (Nation Institutes of Health, Bethesda, MD, USA) for the two time points (D_t=0_ and D_t=24_, respectively). The wound closure ratio was then obtained as (D_t=0_ - D_t=24_)/D_t=0_.

### Transwell assay

The cell migration and invasion assay was performed using Transwells obtained from Corning (For invasion assay, the 8 μm pores of the upper chamber were coated with 50 μg of Matrigel). 10^5^ cells in 0.5ml serum-free medium were placed in the upper chamber. The lower chamber was loaded with 0.8 ml medium containing 10% FBS. After incubating for 36 h, The migrated/invaded cells were stained with DAPI (Santa Cruz, USA, catalog #sc-24941) for 1h at 37℃, and then imaged under a confocal microscope (ZEISS Axio Scan, Germany).

### Evaluation of cell apoptosis

Cell apoptosis was probed using the FITC Annexin V/7-AAD Apoptosis Detection Kit (BD Pharmingen, USA) according to the manufacturer's instructions. The ESCC cells were collected, washed with DPBS and Binding Buffer at 4°C, and resuspended in 200uL of Binding Buffer. A total of 1×10^6^ cells were stained with 2.5uL of Annexin V-FITC and 5uL of 7-AAD. Cells were incubated with the staining solution at room temperature in the dark. Then cells were washed and acquired on a FACScan flow cytometer (BD FACSVerse™, USA).

### Immunofluorescence

Immunofluorescence detection was used to test E-cadherin and N-cadherin on ESCC cells. ESCC cells were plated at a density of 2.5×10^5^ cells per well in 6-well plates with coverslips. The coverslips were washed 3 times with PBS and then fixed in 4% paraformaldehyde before permeabilization with 0.3% Triton X-100. After blocking with 1% BSA (Sigma), cells were incubated with primary antibodies overnight at 4°C, then washed 3 times with PBS. Afterwards they were incubated with the fluorochrome-conjugated secondary antibody for 2h at 37°C. The nuclei were stained with DAPI (Santa Cruz, USA, catalog #sc-24941). Three random fields were examined the Leica TCS SP5 Confocal Laser Scanning Microscope. See supplementary Table 1 for antibody information.

### Statistical analysis

Statistical analyses were performed using GraphPad Prism software (version 5.0). Data were expressed as mean ± SD, and student's t test was used to determine the significance of differences between two groups. P < 0.05 was considered statistically significant.

## Results

### Expression of LRG1 in ESCC

**Fig 1:**
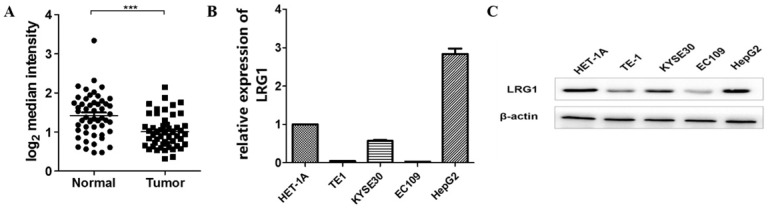
** Expression levels of LRG1 in patients with ESCC and select ESCC cell lines.** (A) Comparison of the mRNA levels of LRG1 in tumor and adjacent normal tissue samples from 51 patients with ESCC. The analysis was done with dataset GSE23400 from the GEO database, and the mRNA levels were reflected by the median centered intensity of the original profiling signal. *** indicates P < 0.001. (B) The mRNA levels of LRG1 in select ESCC cell lines as revealed by real-time PCR. Three randomly chosen ESCC cell lines (TE1, KYSE30 and EC109) were tested along with the normal esophageal tissue cell HET-1A and the *hepatoma* line HepG2 as positive control. The relative mRNA levels of LRG1 in HET-1A was set as 1. (C) The protein levels of LRG1 in the select ESCC cell lines as revealed by Western blot. β-actin was used as loading control.

To determine whether the expression of LRG1 is dysregulated in ESCC, we first analyzed a set of profiling data from the public database GEO (GSE23400), which was derived from 51 ESCC samples and their matched normal tissue 20. The mRNA expression of LRG1 was found to be significantly decreased in patients with ESCC (P < 0.001, Fig. 1A).

To explore if the expression levels of LRG1 were similarly dysregulated in ESCC patient-derived cells, we determined the expression levels of LRG1 in three randomly chosen ESCC cell lines (TE1, KYSE30 and EC109). As shown in Fig. 1B**,** the mRNA and protein levels of LRG1 were consistently decreased in all the three lines tested, as compared to the normal esophageal tissue cell HET-1A, albeit to various extents. Thus the decreased expression of LRG1 in ESCC patients may be well maintained in at least some ESCC cell lines.

### Silencing of LRG1 promoted ESCC cell migration and invasion

**Fig 2:**
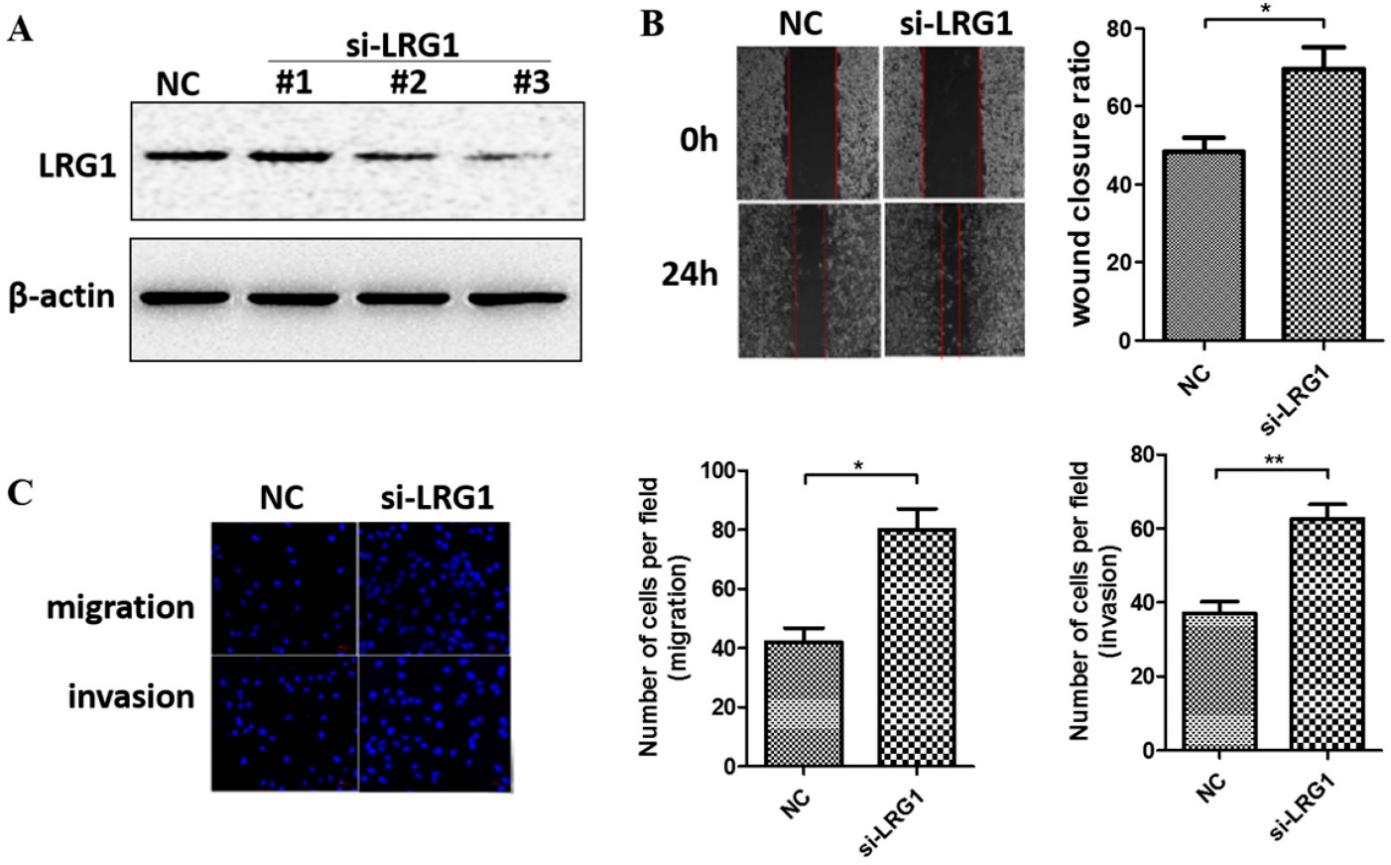
** Effect of LRG1 knockdown on ESCC cell migration and invasion.** (A) Western blot analysis of LRG1 protein levels in KYSE30 cells after transfection of 3 different siRNAs of LRG1 or a scramble negative control (NC). β-actin was used as loading control. NC or siLRG1 #2 transfected cells were starved for 12 h before wound-healing or transwell assay. (B) For wound-healing, cells were manually scratched using a pipette tip, washed and maintained in serum-free culture medium. Pictures were taken at 0 h and 24 h after scratching (left), and the wound closure ratio (cell migration distance at 24 h divided by the gap distance at 0 h) was obtained for comparison (right). Data were representative of three independent experiments and shown as mean ± SD. ** indicates P < 0.01. (C) For transwell assay, cells were added into the upper chamber in serum-free culture medium. The chamber was then placed in a well of 24-well plate that was filled with FBS-containing complete medium. 36 hours later, migrated cells were fixed and stained with DAPI for imaging. The invasion assay was performed similarly except that matrigel coated chambers was used (left). The number of migrated or invaded cells between the two groups were compared (right). Data were representative of three independent experiments and shown as mean ± SD.* indicates P < 0.05, ** indicates P < 0.01.

We next manipulated the expression levels of LRG1 to explore its biological function in ESCC. Given that LRG1 was implicated in cell migration and invasion of other cancer types, we first determined if it also affects these initial steps of metastasis of ESCC. We conducted siRNA interference experiment in KYSE30 cells where the LRG1 expression was relatively high. Two out of three siRNAs (si-LRG1#2 and si-LRG1#3) were found capable of effective knockdown of LRG1 (Fig. 2A) and used in wound-healing and transwell assays. As shown in Fig. 2B, LRG1 knockdown significantly promoted the wound closure of KYSE30 cells (P < 0.01 as compared to the scramble control). Moreover, suppressing the expression of LRG1 enhanced the EACC cells migration and invasive abilities in transwell assays (Fig. 2C, both P < 0.05). In addition, the same stimulatory effects on cell mobility were also observed in TE1 cells upon LRG1 knockdown, providing further validation (Fig. S1). These results provided evidence for a role of LRG1 in inhibition of the migration and invasion of ESCC cells.

### Overexpression of LRG1 inhibited ESCC cell migration and invasion

**Fig 3:**
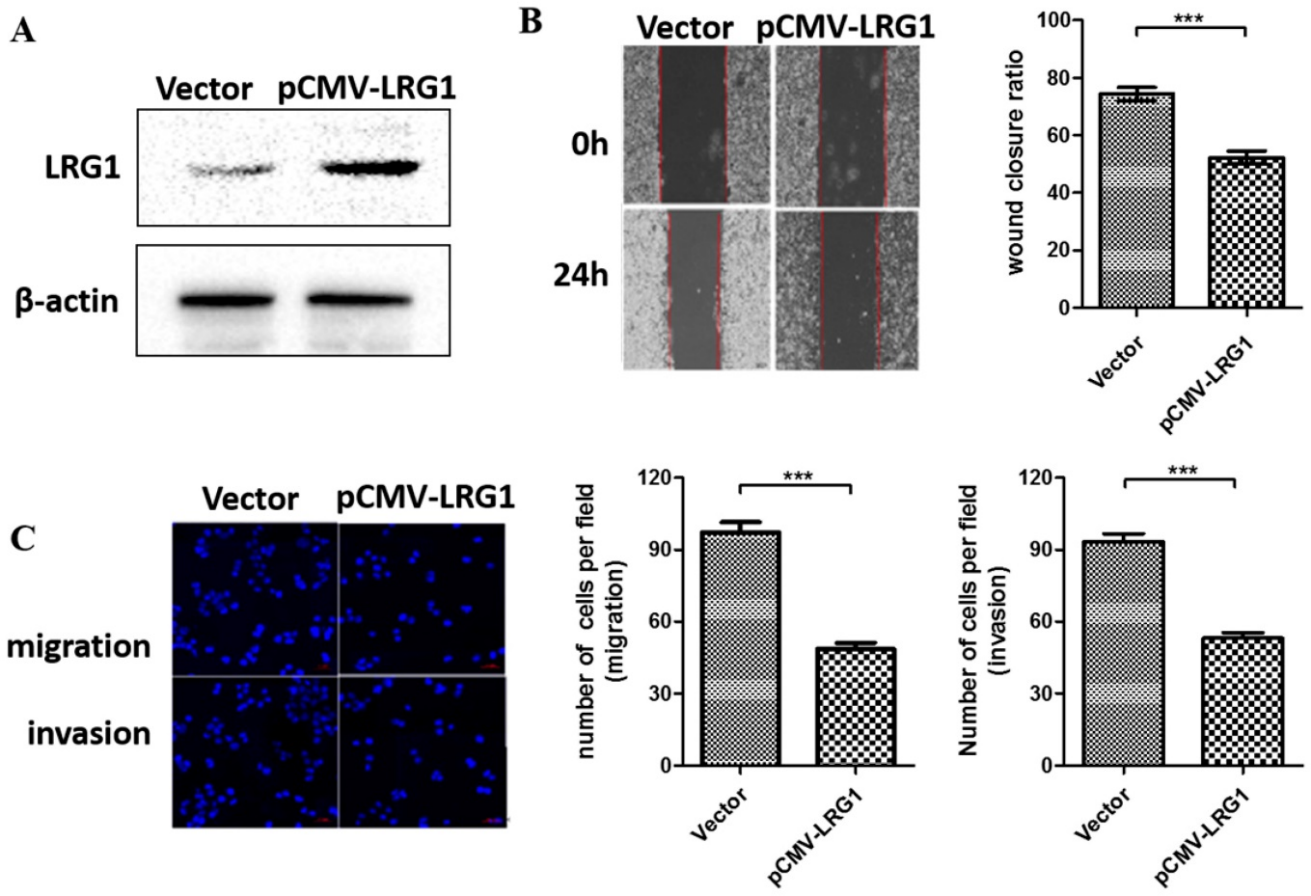
** Effect of LRG1 overexpression on ESCC cell migration and invasion.** (A) Western blot analysis of LRG1 protein levels in EC109 cells after transfection of LRG1-overexpressing plasmid (pCMV-LRG1) or the empty vector. β-actin was used as loading control. Vector or pCMV-LRG1 transfected cells were starved in serum free medium for 12 h before wound-healing or transwell assay. (B) For wound-healing, cells were manually scratched using a pipette tip, washed and maintained in serum-free culture medium. Pictures were taken at 0 h and 24 h after scratching (left), and the wound closure ratio (cell migration distance at 24 h divided by the gap distance at 0 h) was obtained for comparison (right). Data were representative of three independent experiments and shown as mean ± SD.** indicates P < 0.01. (C) For transwell assay, cells were added into the upper chamber in serum-free culture medium. The chamber was then placed in a well of 24-well plate that was filled with FBS-containing complete medium. 36 hours later, migrated cells were fixed and stained with DAPI for imaging. The invasion assay was performed similarly except that matrigel coated chambers was used (left). The number of migrated or invaded cells between the two groups were compared (right). Data were representative of three independent experiments and shown as mean ± SD.*** indicates P < 0.001.

To further examine the function of LRG1 on ESCC cell migration and invasion, we conducted overexpression experiments in EC109 cells where the LRG1 expression were profoundly lost (Fig. 1B). Restoring the expression of LRG1 by transit transfection (Fig. 3A) significantly hindered the migration and invasion of EC109 cells as revealed by both wound healing (Fig. 3B) and transwell assays (Fig 3C).

### Exogenous recombinant LRG1 inhibited ESCC cell migration and invasion

**Fig 4:**
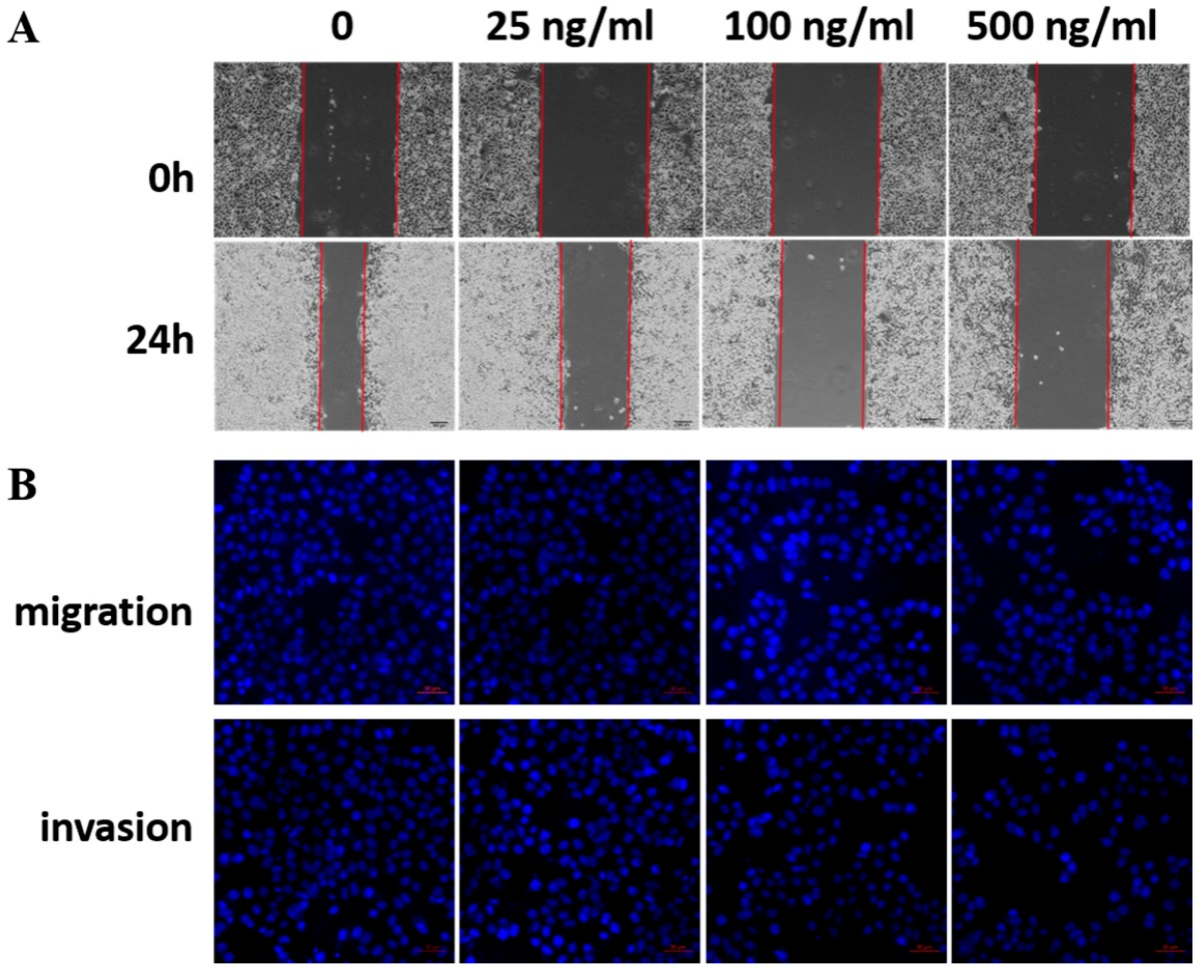
** Effect of recombinant LRG1 on ESCC cell migration and invasion.** EC109 cells were treated with recombinant human LRG1 at indicated concentration and starved in serum free medium for 12 h before wound-healing or transwell assay. (A) For wound-healing, cells were manually scratched using a pipette tip, washed and maintained in serum-free culture medium. Pictures were taken at 0 h and 24 h after scratching. (B) For transwell assay, cells were added into the upper chamber in serum-free culture medium. The chamber was then placed in a well of 24-well plate that was filled with FBS-containing complete medium. 36 hours later, migrated cells were fixed and stained with DAPI for imaging. The invasion assay was performed similarly except that matrigel coated chambers was used. Data were representative of three independent experiments.

LRG1 is a secretory protein. To determine whether LRG1 functions in an autocrine/paracrine manner, we treated EC109 cells with recombinant human LRG1 protein at different concentrations ranging from 0-500 ng/mL for 24h, then conducted scratch test and transwell assays. Indeed, as the concentration of recombinant human LRG1 increased, the migration and invasion ability of EC109 cells decreased (Fig. 4). The same results were obtained when KYSE30 cells were also treated with recombinant LRG1 (Fig. S2), which was further validated in TE1 cells (Fig. S3). Taken together with the knockdown and overexpression results described above, we demonstrated an inhibitory role of LRG1 in ESCC cell migration and invasion.

### LRG1 inhibited ESCC cell metastasis by reducing EMT via TGFβ/SMAD signaling pathway

**Fig 5:**
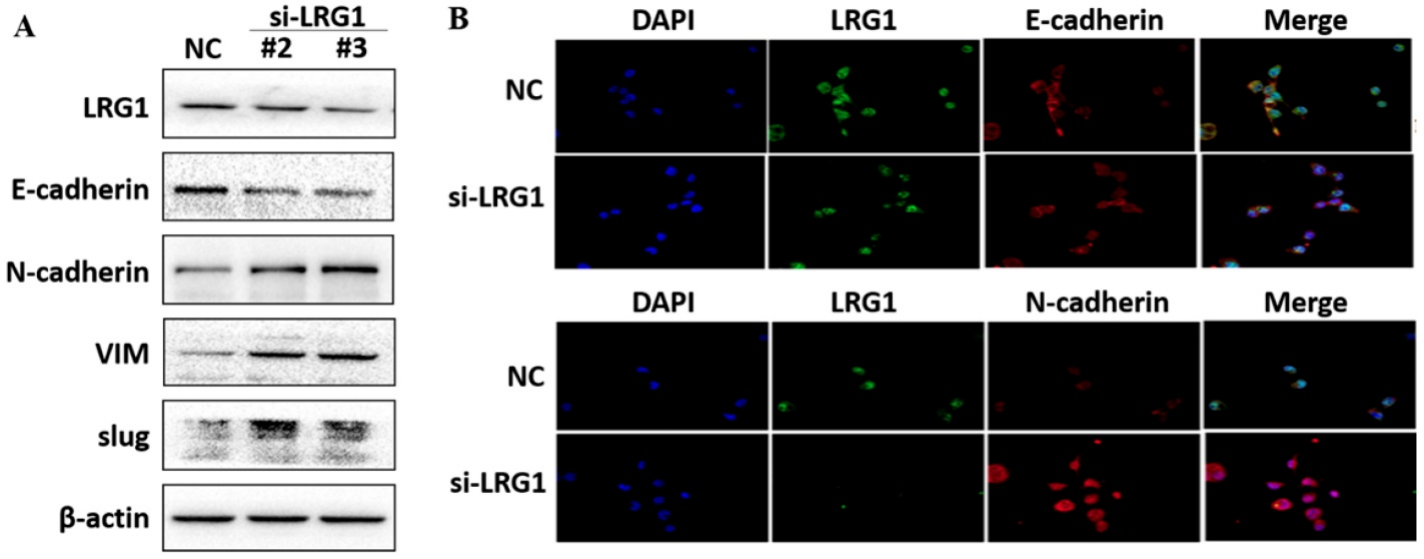
** Effect of LRG1 knockdown on the epithelial to mesenchymal transition (EMT) of ESCC cells.** (A) Western blot analysis of protein expression of EMT marker genes upon LRG1 knockdown in KYSE30 cells with siRNA #2 and #3. β-actin was used as loading control. (B) Immunofluorescence staining of E-cadherin (top) and N-cadherin (bottom) in NC or siLRG1 #2 transfected cells. Data were representative of three independent experiments.

**Fig 6:**
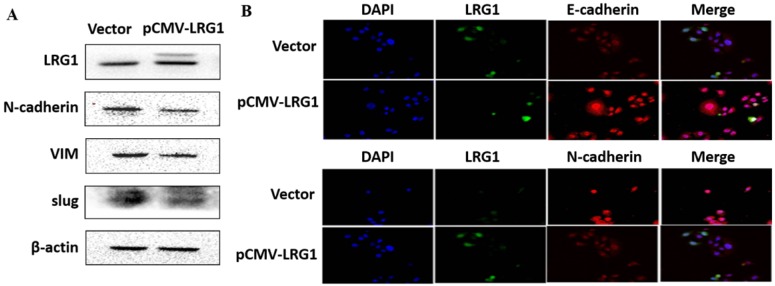
** Effect of LRG1 overexpression on the epithelial to mesenchymal transition (EMT) of ESCC cells.** (A) Western blot analysis of protein expression of EMT marker genes upon LRG1 overexpression in EC109 cells. β-actin was used as loading control. (B) Immunofluorescence staining of E-cadherin (top) and N-cadherin (bottom) in vector or pCMV-LRG1 transfected cells. Data were representative of three independent experiments.

EMT is the biological process by which epithelial cells are transformed into mesenchymal phenotype cells and acquire the ability to migrate and invade 21, 22. This process can downregulate E-cadherin but upregulate N-cadherin, vimentin (VIM) and slug through modulating EMT-related signaling pathways 23, 24. We therefore examined whether enhanced migration and invasion of ESCC cells with LRG1 knockdown was achieved by promoting EMT. Western blotting provided confirmation that knockdown of LRG1 increased the protein levels of N-cadherin, VIM and slug, while reduced that of E-cadherin in KYSE30 cells (Fig. 5A). The corresponding changes in the expression levels of E-cadherin and N-cadherin were also consistently found in the immunofluorescence experiments (Fig. 5B). To provide further evidence, we conducted LRG1 knockdown in TE1 cells observed the same changes (Fig. S4). By contrast, the expression of N-cadherin, VIM and slug was reduced when LRG1 was overexpressed in EC109 cells (Fig. 6A and 6B). While we were not able to detect E-cadherin expression in EC109 cells by Western blotting, we did found a remarkable increase in its levels in LRG1 overexpressed cells by immunofluorescence staining (Fig. 6B). In line with this, treatment of EC109, TE1 and KYSE30 cells with increasing amount of recombinant LRG1 also decreased the protein levels of E-cadherin, but increased that of N-cadherin, VIM and slug (Fig. S5 and S6). Therefore, addition of exogenous of LRG1 protein could suppress the EMT of ESCC cells regardless of differences in endogenous LRG1 levels.

**Fig 7:**
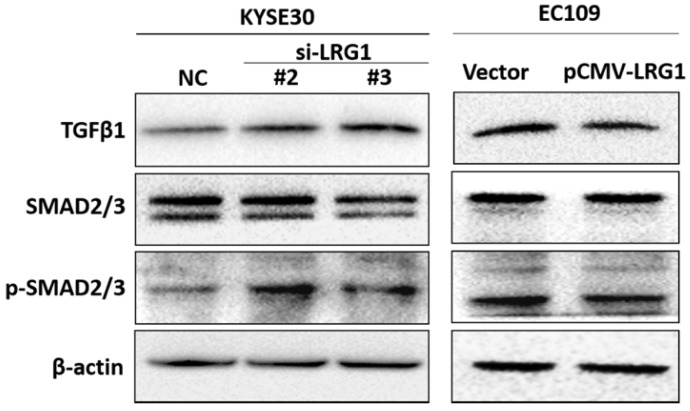
** Manipulation of LRG1 levels modulated the activation of TGFβ pathway.** Shown were western blot analysis of protein expression of TGF-β1, SMAD2/3 and phosphorylated SMAD2/3 upon LRG1 knockdown in KYSE30 cells with siRNA #2 and #3 (left), or upon LRG1 overexpression in EC109 cells (right). β-actin was used as loading control. Data were representative of three independent experiments.

TGF-β/SMAD pathway is involved in tumor formation and progression. Activation of TGF-β/SMAD plays an important role in enhancing the EMT 25-27. We found that knockdown of LRG1 expression in KYSE30 cells could activate the TGF-β/SMAD pathway, as revealed by enhanced levels of TGF-β1 as well as phosphorylated SMAD2/3 (Fig. 7), which was consistently observed in TE1 cells (Fig. S7). By contrast, when LRG1 was ectopically expressed in EC109 cells, TGF-β1 levels and the downstream phosphorylation of SMAD2/3 were reduced (Fig. 7). Together, our findings suggest that LRG1 served as a negative regulator of the TGF-β1/SMAD 2/3 pathway, which impacted the EMT and migration and invasion of ESCC cells.

### LRG1 promoted apoptosis of ESCC cells

Studies have shown that LRG1 could also affect the apoptosis of tumor cells 28, 29. We analyzed the ESCC cell apoptosis by flow cytometry after manipulating LRG1 expression. AS shown in Fig. S8, the number of annexin V positive, 7-AAD staining negative apoptotic cells was markedly decreased in LRG1-silenced KYSE30 cells but significantly increased in LRG1-overexpressed EC109 cells, as compared with their respective negative controls (both P < 0.01). When the protein levels of apoptosis suppressor B-cell lymphoma-2 (Bcl-2) and its downstream protein cleaved caspase-3 and Bax were analyzed, we found that knockdown of LRG1 in KYSE30 up-regulated the amount of Bcl-2, but down-regulated the expression of Bax and cleaved caspase-3 (Fig. S9). By contrast, when LRG1 is overexpressed in EC109 cells, the Bcl-2 protein expression was down-regulated, while Bax and cleaved caspase-3 levels were up-regulated (Fig. S9). Therefore, LRG1 also played a role in promoting apoptosis in ESCC cells. Decreased expression of LRG1 in ESCC might favor tumor growth due to reduced apoptosis.

## Discussion

The prognosis of esophageal squamous cell carcinoma is still poor due to the high chance of recurrence and metastasis. Current studies suggest that many tumor-related genes are involved in the behavioral regulation of cancer cells and play a role in promoting or inhibiting cancer 30, but the molecular mechanism of ESCC invasion and metastasis is still not fully understood 31.

LRG1 has been reported to be highly expressed in gastric cancer, colorectal cancer, brain glioma and other tumors 17, 32, 33. The high expression of LRG1 was found to promote the invasion and migration of those cancer cells. However, it has also been reported that the expression of LRG1 in liver cancer cell lines is lower than that in normal liver cells, and the overexpression of LRG1 could suppress the invasion and migration of liver cancer cells 18. In our study, we first demonstrated that the levels of LRG1 mRNA were significantly decreased in patients with ESCC by mining of the GEO database, which appeared to be well maintained in several patient-derived cell lines. Further, we found that silencing of LRG1 accelerated, while overexpression of LRG1 inhibited, the migration and invasion of ESCC cells. Our data suggested that in ESCC metastasis LRG1 may play a role of tumor suppressor, consistent with previous study by Zhang et al in HCC 18 but on contrary to others 14.

As a secreted protein, some studies have reported that LRG1 is highly expressed in the serum of patients with non-small cell lung cancer 34, oral cancer 35, colon cancer 11, etc. Some studies suggest that LRG1 can be used as a tumor marker or indicator of tumor screening. In our study, we found that treatment of ESCC cells with recombinant human LRG1 protein reduced their motor ability in a LRG1 concentration-dependent manner. Decreased expression of LRG1 in patients with ESCC might therefore lessen an important control of metastasis due to decreased secretion of LRG1 protein that would otherwise work on ESCC cells in an autocrine/paracrine fashion.

Although it has been clear that LRG1 is involved in a variety of malignant diseases, the molecular mechanism of its regulation of tumor has not been fully clarified. Recent studies have shown that LRG1 is involved in cell adhesion, cell migration, cell invasion and pathological angiogenesis of tumor 36. It has also been shown that LRG1 is involved in the epithelial-to-mesenchymal transition (EMT) process 33,37. Our study showed that in ESCC cells E-cadherin was down-regulated, while N-cadherin, VIM and slug were up-regulated upon the silencing of LRG1. The opposite regulation was observed when LRG1 mediated signaling was amplified by either overexpression or treatment with the recombinant protein. This appeared to be achieved via regulation of TGF-β pathway, as manipulating LRG1 expression resulted in corresponding changes in TGF-β1 levels and the downstream phosphorylation of SMAD2/3.

Abnormal apoptosis of cells is closely related to tumorigenesis 38. The silencing of LRG1 has been reported to inhibit cell apoptosis of different cancers 28, 29, yet our study showed that LRG1 may promote apoptosis of ESCC cells as a tumor suppressor. While this manuscript was in preparation, another group reported their study on the expression of LRG1 in a cohort of patients with ESCC that found elevated levels of LRG1 in the tumor tissue 39. In their functional analysis with shRNA knockdown, silencing of LRG1 appeared to suppress invasion while promoting apoptosis of ESCC cells 39. Yet, the report did not show any data from gain-of-function studies (by either overexpression or addition of recombinant of LRG1, as we did here) to see whether opposite effects could be observed. It is not clear whether disease stage and/or treatment affect LRG1 expression in different ESCC patients. To better understand the clinical correlation, further studies with enlarged sample size are required to determine the aberrant expression of LRG1 in ESCC. Also, although we achieved the same conclusion using three different ESCC cell lines, to demonstrate how our laboratory findings would reflect the in vivo status, animal experiments such as xenograft test are warranted to provide solid evidence on the impact and mechanisms of LRG1 on ESCC. Nevertheless, our data suggest that LRG1 might play multiple roles in tumor formation and metastasis of ESCC. And given the relatively easy access nature of secretary proteins, LRG1 might serve as a novel therapeutic target for ESCC.

## Supplementary Material

Supplementary figures and table.
