# Supplementary material for: LRG1 Suppresses Migration and Invasion of Esophageal Squamous Cell Carcinoma by Modulating Epithelial to Mesenchymal Transition
Source: J Cancer. 2020 Jan 14;11(6):1486–94. doi: 10.7150/jca.36189 (PMC6995366; doi:10.7150/jca.36189)
Supplement: Supplementary file 1 — Supplementary figures and table. [file jcav11p1486s1.pdf]

Supplementary Table 1. Antibody list.

Antibody	Supplier	Catalog #
LRG1	Abcam	ab170953
E-Cadherin	Cell Signaling Technology	3195
N-Cadherin	Cell Signaling Technology	13116
Vimentin	Cell Signaling Technology	5741
Slug	Cell Signaling Technology	9585
Smad2/3	Cell Signaling Technology	8685
Phospho-Smad2/3	Cell Signaling Technology	8828
Bcl-2	Cell Signaling Technology	15071
Bax	Cell Signaling Technology	14796
Cleaved Caspase-3	Cell Signaling Technology	9664
β-Actin	Cell Signaling Technology	4970
TGFβ1	ImmunoWay	YT4632
Mouse Anti-rabbit IgG mAb (HRP Conjugate)	Cell Signaling Technology	5127
Anti-mouse IgG, HRP-linked Antibody	Cell Signaling Technology	7076
Alexa Fluor ® 568 goat anti-rabbit IgG	Thermo Fisher	A-11011
Alexa Fluor ® 594 goat anti-mouse IgG	Thermo Fisher	A-11015

Supplementary Figure Legends.

Fig. S1. Effect of LRG1 knockdown on TE1 cells migration and invasion. (A) Western blot analysis of LRG1 protein levels in TE1 cells after transfection of 3 different siRNAs of LRG1 or a scramble negative control (NC). β-actin was used as loading control. NC or siLRG1 #2 transfected cells were starved for 12 h before wound-healing or transwell assay. (B) For wound-healing, cells were manually scratched using a pipette tip, washed and maintained in serum-free culture medium. Pictures were taken at 0 h and 24 h after scratching (left), and the wound closure ratio (cell migration distance at 24 h divided by the gap distance at 0 h) was obtained for comparison (right). Data were representative of three independent experiments and shown as mean ± SD. (C) For transwell assay, cells were added into the upper chamber in serum-free culture medium. The chamber was then placed in a well of 24-well plate that was filled with FBS-containing complete medium. 36 hours later, migrated cells were fixed and stained with DAPI for imaging. The invasion assay was performed similarly except that matrigel coated chambers was used (left). The number of migrated or invaded cells between the two groups were compared (right). Data were representative of three independent experiments and shown as mean ± SD. ** indicates $P < 0.01$, *** indicates $P < 0.001$.

Fig. S2. Effect of recombinant LRG1 on KYSE30 cell migration and invasion. KYSE30 cells were treated with recombinant human LRG1 at indicated concentration and starved in serum free medium for 12 h before wound-healing or transwell assay. (A) For wound-healing, cells were manually scratched using a pipette tip, washed and maintained in serum-free culture medium. Pictures were taken at 0 h and 24 h after scratching. (B) For transwell assay, cells were added into the upper chamber in serum-free culture medium. The chamber was then placed in a well of 24-well plate that was filled with FBS-containing complete medium. 36 hours later, migrated cells were fixed and stained with DAPI for imaging. The invasion assay was performed similarly except that matrigel coated chambers was used. Data were representative of three independent experiments.

Fig. S3. **Effect of recombinant LRG1 on TE1 cells migration and invasion**. TE1 cells were treated with recombinant human LRG1 at indicated concentration and starved in serum free medium for 12 h before wound-healing or transwell assay. (A) For wound-healing, cells were manually scratched using a pipette tip, washed and maintained in serum-free culture medium. Pictures were taken at 0 h and 24 h after scratching. (B) For transwell assay, cells were added into the upper chamber in serum-free culture medium. The chamber was then placed in a well of 24-well plate that was filled with FBS-containing complete medium. 36 hours later, migrated cells were fixed and stained with DAPI for imaging. The invasion assay was performed similarly except that matrigel coated chambers was used. Data were representative of three independent experiments.

Fig. S4. Effect of LRG1 knockdown on the epithelial to mesenchymal transition (EMT) of TE1 cells. (A) Western blot analysis of protein expression of EMT marker genes upon LRG1 knockdown in TE1 cells with siRNA #2 and #3. β-actin was used as loading control. (B) Immunofluorescence staining of E-cadherin (top) and N-cadherin (bottom) in NC or siLRG1 #2 transfected cells. Data were representative of three independent experiments.

Fig. S5. Effect of recombinant LRG1 on the epithelial to mesenchymal transition (EMT) of ESCC cells. KYSE30 and EC109 cells were treated with recombinant human LRG1 at indicated concentration for 24 h. Cells were then harvested for western blot

analysis of protein expression of indicated EMT marker genes. β-actin was used as loading control. Data were representative of three independent experiments.

Fig. S6. Effect of recombinant LRG1 on the epithelial to mesenchymal transition (EMT) of TE1 cells. TE1 cells were treated with recombinant human LRG1 at indicated concentration for 24 h. Cells were then harvested for western blot analysis of protein expression of indicated EMT marker genes. β-actin was used as loading control. Data were representative of three independent experiments.

Fig. S7. Effect of LRG1 knockdown on the activation of TGFβ pathway in TE1 cells. Shown were western blot analysis of protein expression of TGF-β1, SMAD2/3 and phosphorylated SMAD2/3 upon LRG1 knockdown in TE1 cells with siRNA #2 and #3. β-actin was used as loading control. Data were representative of three independent experiments.

Fig. S8. Manipulation of LRG1 levels affected ESCC cell apoptosis. Shown were flow cytometry analysis of cell apoptosis with Annexin V and 7-AAD staining upon LRG1 knockdown in KYSE30 cells with siRNA #2 (top), or upon LRG1 overexpression in EC109 cells (bottom). Apoptosis ratio of LRG1 silenced or overexpressed cells were compared to that of scramble NC or empty vector transfected cells, respectively. Data were representative of three independent experiments and shown as mean ± SD. ** indicates $P < 0.01$, *** indicates $P < 0.001$.

Fig. S9. Manipulation of LRG1 levels modulated the activation of apoptosis pathway in ESCC cells. Shown were western blot analysis of protein expression of Bcl-2, Bax and cleaved caspase 3 upon LRG1 knockdown in KYSE30 cells with siRNA #2 and #3 (left), or upon LRG1 overexpression in EC109 cells (right). β-actin was used as loading control. Data were representative of three independent experiments.

Fig. S1

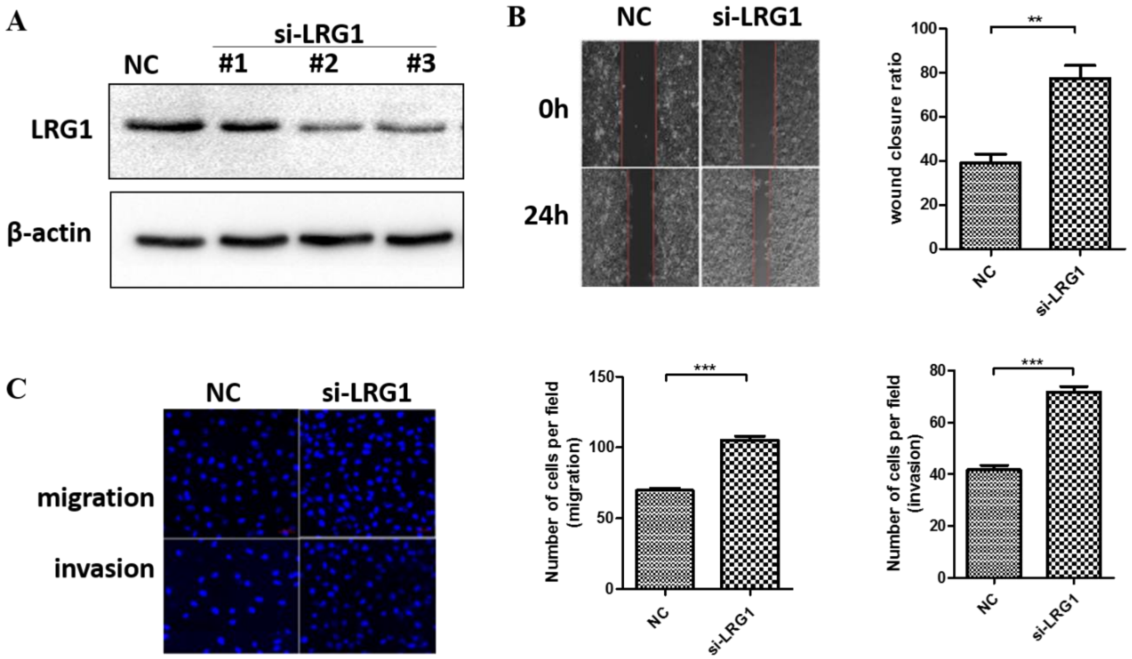
A
si-LRG1
NC
#1
#2
#3
LRG1
β-actin
B
NC
si-LRG1
0h
24h
**
wound closure ratio
100
80
60
40
20
0
NC
si-LRG1
C
NC
si-LRG1
migration
invasion
***
Number of cells per field (migration)
150
100
50
0
NC
si-LRG1
***
Number of cells per field (invasion)
80
60
40
20
0
NC
si-LRG1

Fig. S2

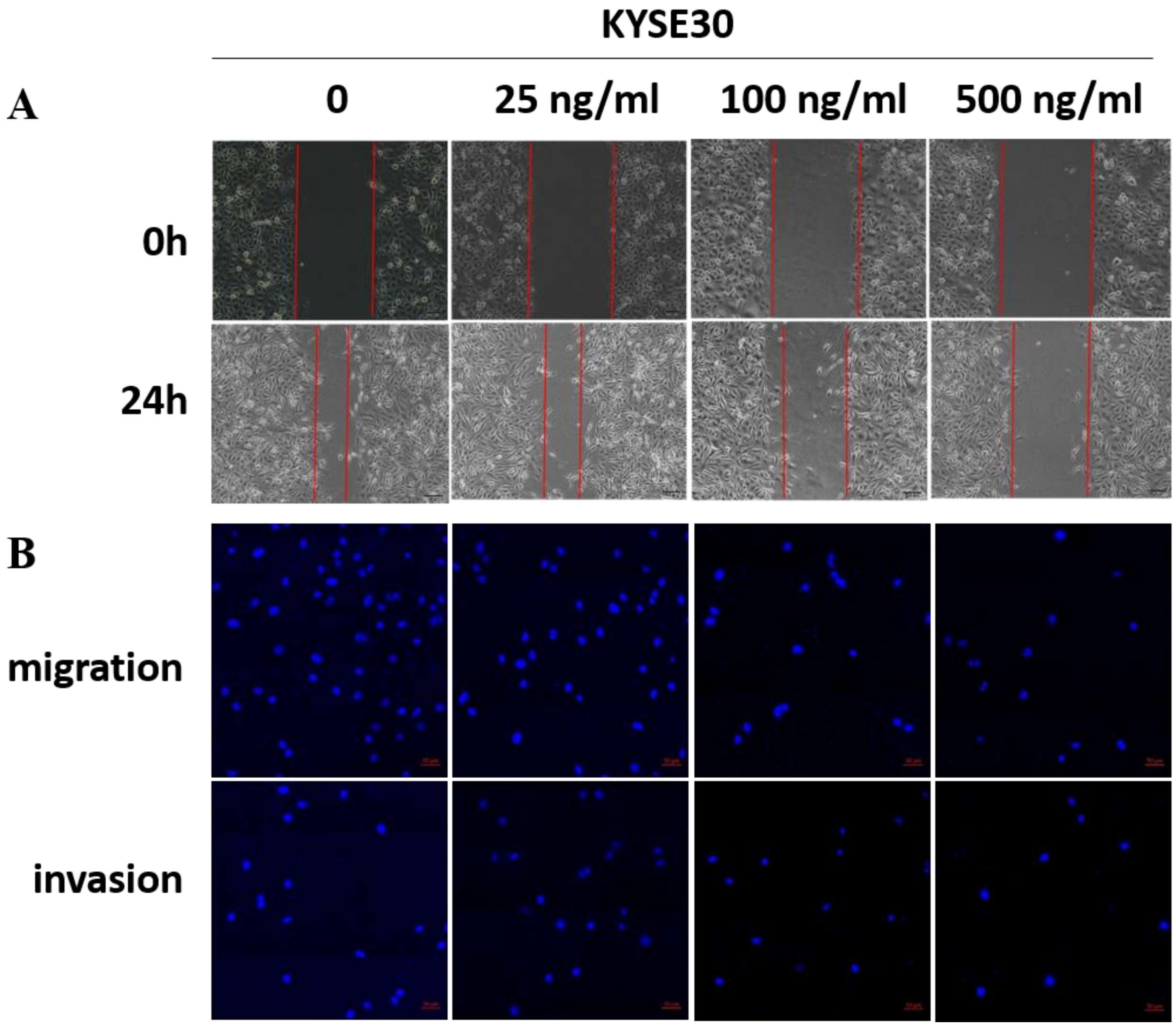

KYSE30
A
0
25 ng/ml
100 ng/ml
500 ng/ml
0h
24h
B
migration
invasion

Fig. S3

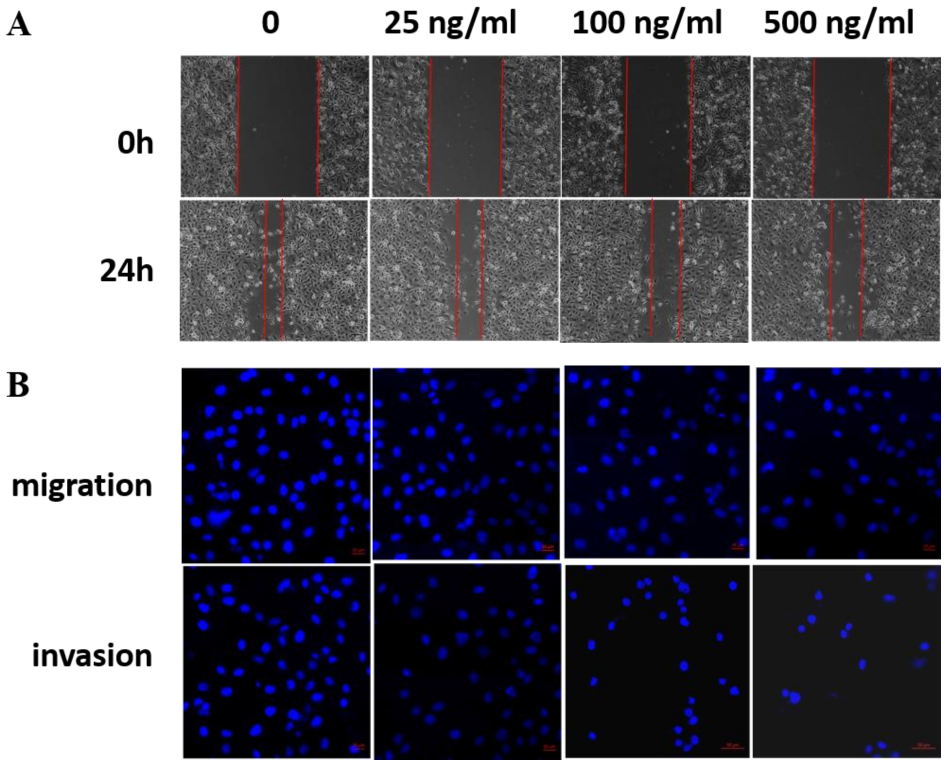

Fig. S4

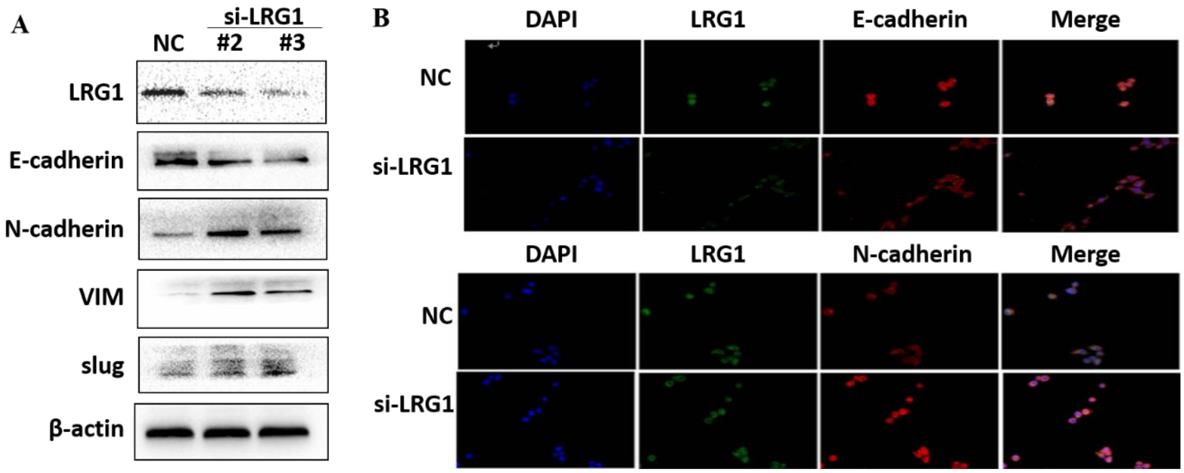

A
si-LRG1
NC
#2
#3
LRG1
E-cadherin
N-cadherin
VIM
slug
β-actin
B
DAPI
LRG1
E-cadherin
Merge
NC
si-LRG1
DAPI
LRG1
N-cadherin
Merge
NC
si-LRG1

Fig. S5

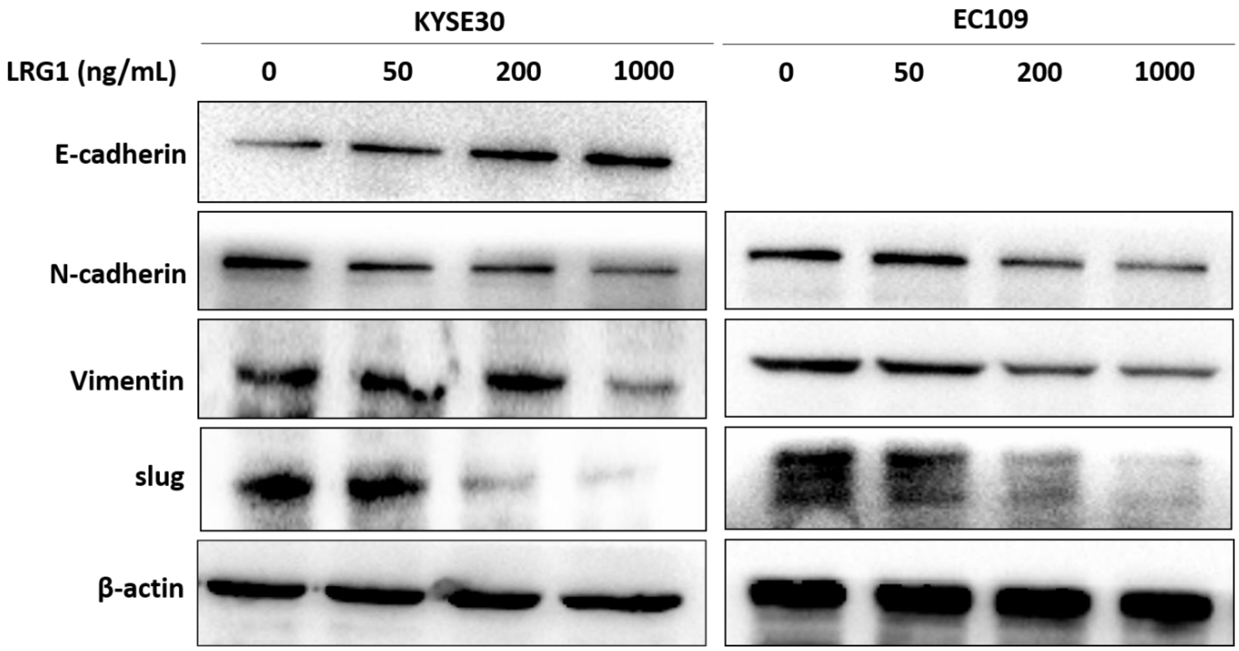
KYSE30
EC109
LRG1 (ng/mL)
0
50
200
1000
0
50
200
1000
E-cadherin
N-cadherin
Vimentin
slug
β-actin

Fig. S6

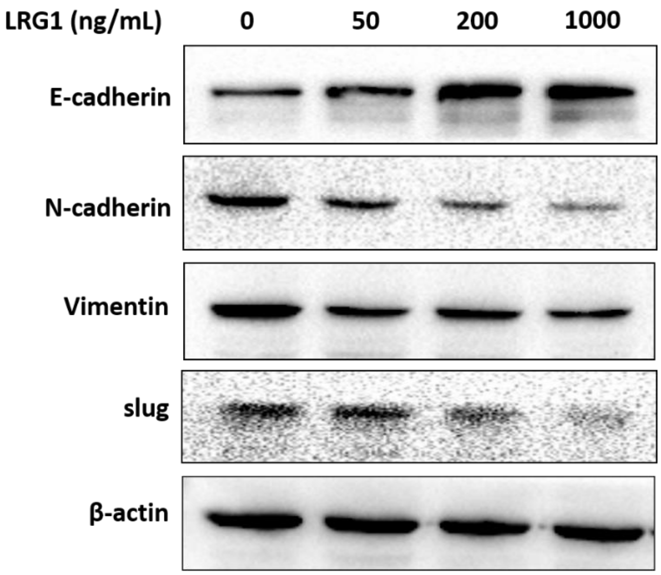
LRG1 (ng/mL) 0 50 200 1000
E-cadherin
N-cadherin
Vimentin
slug
β-actin

Fig. S7

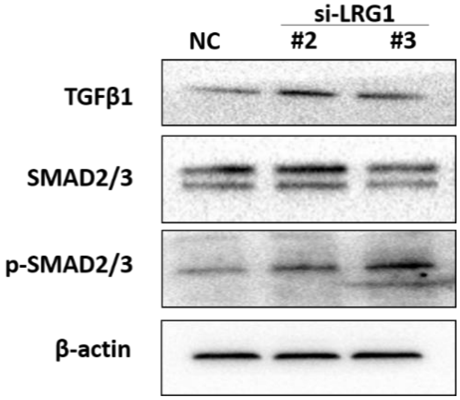

Fig. S8

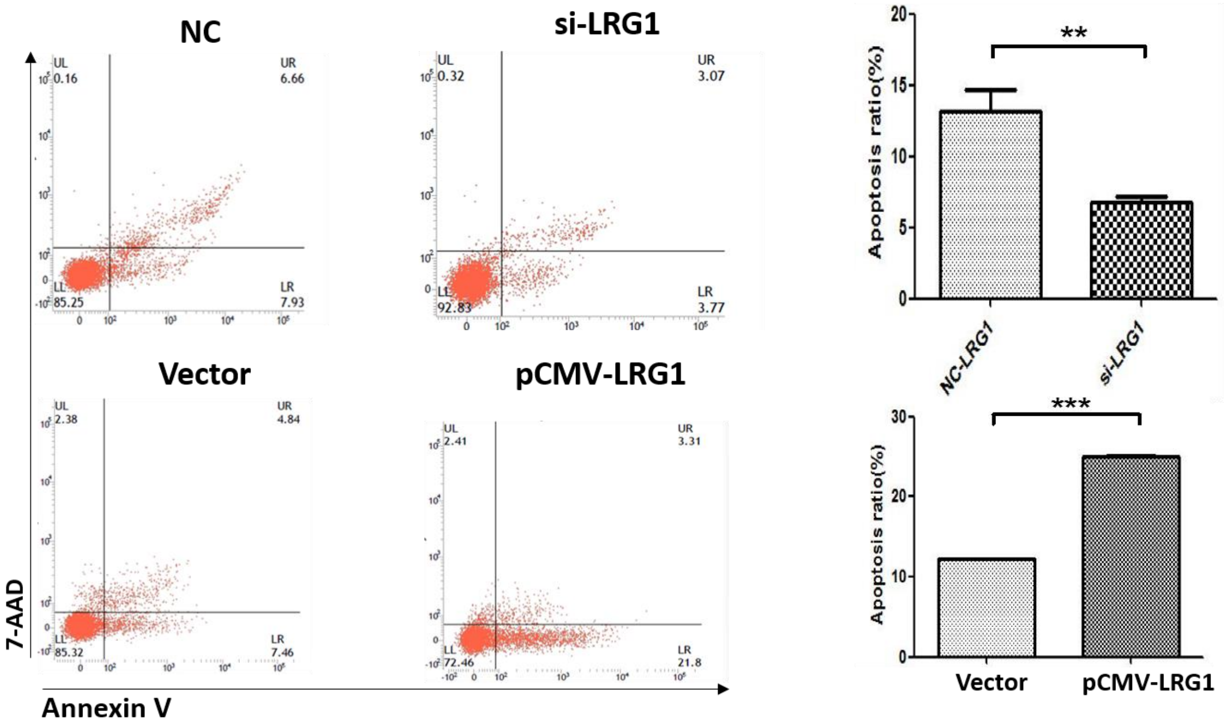

NC
si-LRG1
Vector
pCMV-LRG1
UL 0.16
UR 6.66
LL 85.25
LR 7.93
UL 0.32
UR 3.07
LL 92.83
LR 3.77
UL 2.38
UR 4.84
LL 85.32
LR 7.46
UL 2.41
UR 3.31
LL 72.46
LR 21.8
7-AAD
Annexin V
Apoptosis ratio(%)
**
NC-LRG1
si-LRG1
***
Vector
pCMV-LRG1

Fig. S9

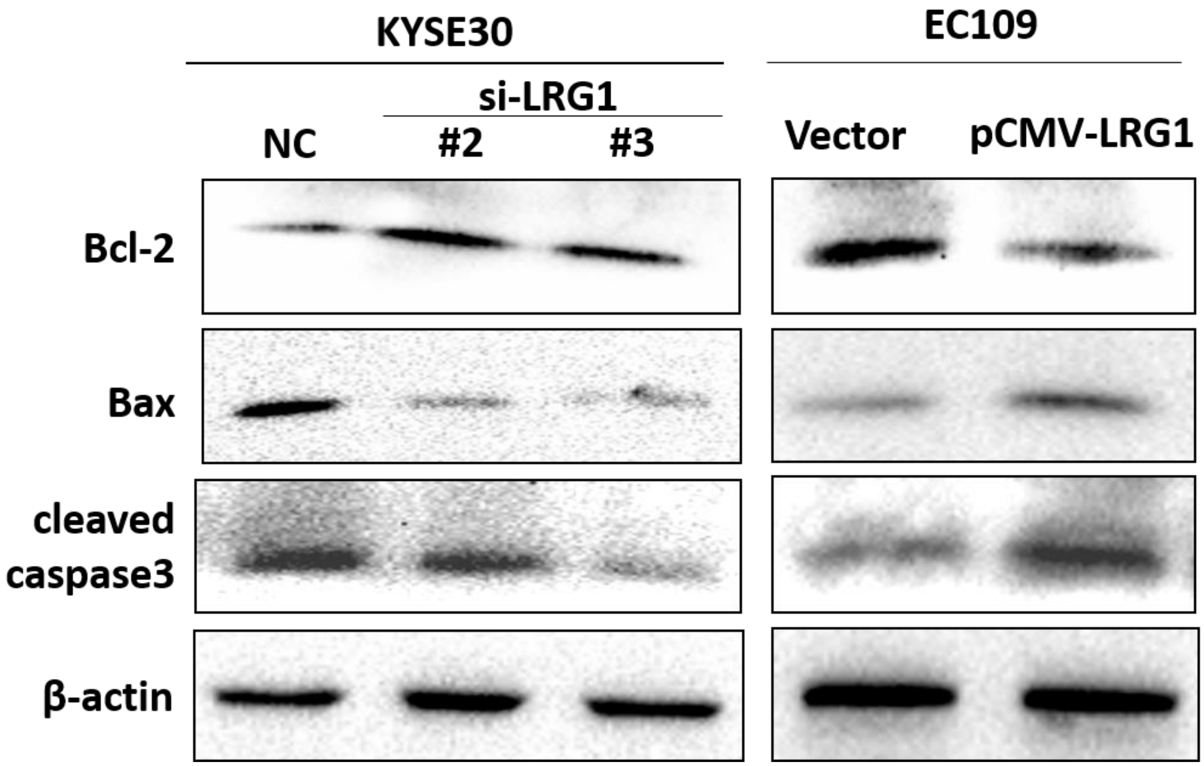
KYSE30
EC109
si-LRG1
NC
#2
#3
Vector
pCMV-LRG1
Bcl-2
Bax
cleaved caspase3
β-actin
